# Tetralogy of Fallot repair in patients presenting after Infancy: A single surgeon experience

**DOI:** 10.12669/pjms.334.12891

**Published:** 2017

**Authors:** Tariq Waqar, Muhammad Usman Riaz, Tania Mahar

**Affiliations:** 1 Tariq Waqar, MBBS, FCPS, FRCS. Associate Professor of Pediatric Cardiac Surgery, CPE Institute of Cardiology, Multan, Pakistan; 2 Muhammad Usman Riaz, FCPS. Senior Registrar, Pediatric Cardiac Surgery CPE Institute of Cardiology, Multan, Pakistan; 3 Tania Mahar, FCPS. Registrar, Pediatric Cardiac Surgery, CPE Institute of Cardiology, Multan, Pakistan

**Keywords:** Late repair, Operative mortality, Tetralogy of Fallot

## Abstract

**Objective:**

To determine the early surgical outcomes of Tetralogy of Fallot (TOF) repair in children and young adults operated after the age of one year.

**Methods:**

In this retrospective study, 307 cases of primary repair of Tetralogy of Fallot were done between September 2012 to February 2017, at CPE Institute of cardiology, Multan. Out of 307 operated patients, 4 (1.3%) patients had previous modified Blalock Taussig shunts, 2 (0.6%) associated ASD with TOF, 3 (0.9%) co-association of TOF with PDA, 2 (0.6%) had large conal arterial branch crossing the annulus, 3 (0.9%) had dextrocardia with situs inversus, 12 (3.9%) TOF with double outlet right ventricle (DORV), 2 (0.6%) were associated with complete AV canal defect, 8 (2.60%) with absent pulmonary valve syndrome, 15 (5.5%) with left pulmonary artery stenosis. Data of post-operative complications and operative parameters was recorded for all patients.

**Results:**

Mean age of operated patients was 9.56±4.89 years. Post-operative complications occurred in 7.8% of patients. Most common post-operative complications were pleural effusion with a frequency of 12(3.9%) patients, and complete heart block in one patient. Insignificant small residual VSD was diagnosed in 8 (2.6%) patients. One moderately large VSD was closed surgically after one year of 1^st^ surgery. Moderate to severe pulmonary valve regurgitation was diagnosed in 114 (37.1%) patients. Mild to moderate tricuspid regurgitation in 15 (4.8%) patients and moderate right ventricular outflow tract obstruction (RVOT) in 16 (5.2%) patients. Thirty-day mortality was only four (1.3%).

**Conclusion:**

Surgical correction of Tetralogy of Fallot (TOF) in children after one year carries good operative outcomes with minimum morbidity and mortality.

## INTRODUCTION

Tetralogy of Fallot (TOF) accounts for 7-10% of all congenital cardiac defects and has only 24% 10-year survival rate if left untreated.^1^,^2^ Surgical repair of TOF has evolved over the decades after first creation of systemic to pulmonary shunt in 1944 by Alfred Blalock, followed by complete repair in 1950s by C. Walton Lillehei.^2^,^3^ Complete surgical repair during infancy or neonatal period is considered the best time for surgery.^4^-^6^ The advantages of early repair include; prevention of end-organ damage due to cyanosis, preserved myocardium, prevention of right ventricular hypertrophy, fibrosis and in some cases failure by removing the culprit stimulus and improved development of pulmonary arteries and lungs.^7^ The reported mortality rate is 3.0% in children and up to 9.0% in adults.^5^,^8^-^10^

In developing countries like Pakistan, diagnosis of TOF is often delayed because of limited number of pediatric cardiology and pediatric cardiac surgery departments. Moreover, most of the patients of TOF are diagnosed and treated in later stages of their life, usually after the age of one year. Surgical repair of TOF in older children is associated with more operative complications e.g. bleeding, RV dysfunction, low cardiac output syndrome, arrhythmias and even mortality.^11^ This study is a retrospective analysis of surgical outcomes of TOF in children operated after the age of one year.

## METHODS

In this retrospective analysis, we have shared our experience of surgical repair of TOF of 307 children operated in Ch. Pervaiz Elahi Institute of Cardiology from September 2012 to February 2017. Academic Affairs Department of CPE Institute of Cardiology, Multan approved the study protocol. Data was retrieved from the database of cardiac surgery unit of the institute. No informed consent was taken from guardians of the patients because of the retrospective nature of the study.

Out of 307 operated patients, 4 (1.3%) patients had previous modified Blalock Taussig shunts, 2 (0.6%) associated ASD with TOF, 3 (0.9%) had co-association of PDA, 2 (0.6%) large conal arterial branch crossing at the annulus, 3 (0.9%) dextrocardia with situs inversus, 12(3.9%) TOF with double outlet right ventricle (DORV), 2 (0.6%) with associated complete AV canal defect, 8 (2.60%) with absent pulmonary valve syndrome, 15 (5.5%) with left pulmonary artery stenosis.

A single pediatric cardiac surgeon (First Author) performed all surgeries through median sternotomy and after establishing cardio-pulmonary bypass at moderate hypothermia. Blood cardioplegia was used to arrest the heart after application of cross-clamp. In 45 (14.6%) patients, surgical repair was done through trans-atrial approach. In rest of cases, we used a combined approach (trans-atrial plus trans-pulmonary). Infundibular resection was done in all patients. Pulmonary valvotomy with transannular extension was done in patients with small pulmonary valve annulus. In cases of doubly committed sub-arterial ventricular septal defect (DCSA VSD), VSD closure was done by a combined approach through right atrium and pulmonary artery. In patients with left pulmonary artery stenosis, left pulmonary artery was augmented with autologous pericardial patch. In patients with narrowing of pulmonary annulus, trans-annular pericardial patch was applied. Out of two Patients with large conal branch crossing annulus of PV, repair was done with limited transanular extension in one patient and resection of RVOT through right atrium in another.

In double-outlet right ventricle (DORV) cases, intra-ventricular tunnel was created by using PTFE graft to commit the left ventricle to aorta. In patients with absent pulmonary valve, pulmonary arteries reduction plasty were done along with pulmonary valve placement by inserting bioprosthetic valve of appropriate size.

In patients of TOF with complete AV canal defect, AV canal was repaired using two patch technique along with routine repair of TOF.

Criteria for classification of severity of RVOT was;

**Mild RVOT**: pressure gradient 20-40 mmHg

**Moderate RVOT**: pressure gradient 41-70 mmHg

**Severe RVOT**: pressure gradient >70 mmHg.

Death within 30 day after surgery was labelled as operative mortality. Detailed Morphology of pulmonary valve is given in Table-I. SPSS v23 was used to calculate frequencies for qualitative variables and mean along with standard deviation for quantitative variables.

**Table-I:** Morphology of Pulmonary Valve.

*Variable*	*Frequency (Percentage)*
Normal	59(19.2%)
Commissural stenosis	126 (41.0%)
Small annulus leading to trans annular extension	114 (33.5%)
TOF with absent Pulmonary Valve	8 (2.6%)

## RESULTS

Out of 307 patients, 230 (74.9%) were male. Mean age of operated patients was 9.56±4.89 years. Peri-membranous VSD was predominant and present in 260 (84.6%) patients (Table-II).

**Table-II:** Baseline Characteristics.

*Variable*	*Value*
Age	9.56±4.89
Male gender	230(74.9%)
Female gender	77(25.1%)
Weight	22.74±18.00
***Type of VSD***	
Peri-membranous	260 (84.6%)
DCSA	22 (7.16%)
Outlet VSD	11 (3.58%)
DORV Setting	12(3.90%)
VSD in CAV canal	2(0.65%)
***CPB Characteristics***	
Cardio-pulmonary Bypass time (mins)	145.84±33.3
Cross-clamp time (mins)	106.5±27.49

**Table-III:** Post-Procedural Characteristics.

*Variable*	*Value*
Ventilation time (hours)	7.62±8.46
ICU stay (hours)	44.12±29.85
Inotropic support duration (hours)	27.60±37.61
Hospital stay (days)	7.57±3.80
***Post-operative Complications***	
Pleural effusion	12 (3.9%)
Pneumothorax	5 (1.6%)
Pericardial Effusion	3 (0.97%)
Re-opening due to bleeding	3 (0.97%)
Complete heart Block	1 (0.3%)
Residual VSD	10 (3.25%)
Trivial to small	9 (2.93%)
Moderate	1 (0.32%)
Residual PR	114 (37.1%)
Moderate	51 (16.6%)
Severe	63 (20.5%)
Residual TR	15 (4.8%)
Moderate	15 (4.8%)
Severe	00 (0.0%)
Residual RVOT	16 (5.2%)
Moderate	16 (5.2%)
Severe	0.0
Operative Mortality	4 (1.3%)

Post-operative complications occurred in 7.8% patients. Most common post-operative complication was pleural effusion that occurred in 12 (3.9%) patients, and complete heart block that occurred in one patient in whom permanent pacemaker was inserted.

Residual VSD was diagnosed in 10 (3.25%) patients. Out of these 10, 9 VSDs were tiny or small and one was moderate. One moderate VSD was closed surgically after one year of 1^st^ surgery.

Moderate to severe pulmonary valve regurgitation was observed in 114 (37.1%) patients. Moderate tricuspid regurgitation in 15 (4.8%) patients and moderate right ventricular outflow tract obstruction in 16 (5.2%) patients. No postoperative left ventricular outflow obstruction was observed in patients with DORV repair.

Only 4 (1.3%) patients expired within 30 days after surgery, 2 (0.6%) of these deaths were due to severe post-operative RV dysfunction, one due to post-operative pneumonia and one due to FFP transfusion related reaction. These patients are under follow up of pediatric cardiologists for onset of cardiac symptoms and any change in echocardiographic finding regarding RV function or RVOT gradient.

## DISCUSSION

Tetralogy of Fallot (TOF) in grown up children is still a common presentation in developing countries like Pakistan but in developed countries, it has now become a very rare presentation. And most of the patients of TOF are operated below the age of one year and even at six months. In this study, we have presented our results of repair of TOF in patients of age more than one year at the time of surgery.

Vohra et al. concluded that results of late repair of TOF are comparable to early surgery but late repair is associated with increased intensive care unit stay, mechanical ventilation time and inotropic requirements.^12^ Cabral et al. reported the operative result of TOF after one year of age with operative mortality of 9.0%.^9^ Khan et al. reported 8.0% mortality rate in late TOF patients.^13^ In our study, the operative mortality was only 1.3% which was very low as compared to these studies. Wu et al. reported the results of 212 patients who underwent TOF repair having age 1.5 year to 37 years. These authors reported only 0.9% operative mortality.^14^ In another study by Ghavidel et al the reported operative mortality was 1.9%.^15^ Operative mortality in our study was comparable to the results of these studies. Benbrik et al. reported 4.2% operative mortality rate in TOF children from developing countries.^16^ Dittrich et al. reported very high operative mortality rate of 16% in TOF repair patients having age 18-55 years.^17^ This mortality rate was very high as compared to the other available literature. The main reason for these differences in mortality rate is not known but it may be due to experience of the operating surgeons. Because the survival rate also depends upon the experience and surgical techniques of the operating surgeons.

In our study, the mean duration of ICU stay was 44.12+29.85 hours. In the study of Cabral et al. mean ICU stay of patients was five days,^9^ this time was higher as compared to our study. Alizadeh et al. reported mean ICU stay time of 3.2±1.4 days.^15^ This time was comparable to the ICU stay in our study subjects.

Regarding post-operative complications, the most common complication was pulmonary regurgitation (PR), the incidence of moderate to severe PR was (37.1%). In a study by Khan et al., postoperative incidence of PR was 25%.^13^ In our study, moderate RVOT obstruction after surgery was diagnosed in 5.2% patients. Post-operative pleural effusion occurred in 3.9% patients and complete heart block in only 0.3% patients. In the study by Khan et al., incidence of pericardial effusion was 3.75% and complete heart block in 5.0% patients.^13^ Alizadeh et al. reported 1.9% incidence of complete heart block after TOF repair. Benbrik reported that the complications rate after TOF repair in children from developing countries is high as compared to the patients of developed countries.^16^ In their study the mean age of patients from developing countries was 4.8 years at the time of surgery. In our study, the mean age of patients at the time of surgery was 9.56 years and incidence of post-operative complications were not very high and was comparable with the available literature.

The results of our study are comparable to the results of other international studies. And from these studies we concluded that late repair of TOF after the age of one year results in good outcomes and hence improved quality of life in these patients.

### Limitations

The main limitation is the descriptive design of this study because we did not have the data to compare our results with TOF repair in patients having age less than 1 year. However, we have compared our results with international studies to check the adequacy of the outcomes of our study.

## CONCLUSION

Surgical correction of Tetralogy of Fallot (TOF) in children after one year carries good operative outcomes with minimum morbidity and mortality.

### Author’s Contribution

**TW:** Conceived, designed the research methodology, prepared this manuscript and is accountable for the originality of the research work.

**MUR and TM:** Did data analysis, helped in writing the manuscript and reviewed the manuscript.
